# Microwave assisted synthesis, antifungal activity, DFT and SAR study of 1,2,4-triazolo[4,3-a]pyridine derivatives containing hydrazone moieties

**DOI:** 10.1186/s13065-016-0196-6

**Published:** 2016-08-04

**Authors:** Jin-Xia Mu, Yan-Xia Shi, Hong-Ke Wu, Zhao-Hui Sun, Ming-Yan Yang, Xing-Hai Liu, Bao-Ju Li

**Affiliations:** 1 Department of Environmental Engineering, China Jiliang University, Hangzhou, 310018 Zhejiang China; 2 College of Chemical Engineering, Zhejiang University of Technology, Hangzhou, 310014 China; 3 Institute of Vegetables and Flowers, Chinese Academy of Agricultural Sciences, Beijing, 100014 China

**Keywords:** 1,2,4-Triazolo[4,3-a]pyridine, Hydrazone, Microwave assisted synthesis, Antifungal activity, DFT

## Abstract

**Background:**

The increasing prevalence of multi-drug resistant fungal infections has encouraged the search for new antifungal agents. Hydrazone derivatives always exhibited diversity activities, including antifungal, anti-inflammatory, anti-oxidation, anti-cancer activity. Regarding the heterocyclic moiety, 1,2,4-triazolo[4,3-a]pyridine derivatives also display broad activities, such as antifungal activity, anticonvulsant activity, herbicidal activity, antimicrobial activity and anticancer activity.

**Results:**

A series of novel 1,2,4-triazolo[4,3-a]pyridine derivatives containing hydrazone moiety were designed and synthesized from 2,3-dichloropyridine, hydrazine hydrate by multi-step reactions under microwave irradiation condition, and their structures were characterized by FT IR, ^1^H NMR, ^13^C NMR, ^19^F NMR, MS and elemental analysis. The antifungal activities of title compounds were determined. The results indicated that some of the title compounds exhibited good antifungal activity. Furthermore, DFT calculation was carried out for studying the structure–activity relationship (SAR).

**Conclusion:**

A practical synthetic route to obtain 1,2,4-triazolo[4,3-a]pyridine derivatives is presented. This study suggests that the 1,2,4-triazolo[4,3-a]pyridine derivatives exhibited good antifungal activity.

## Background

Nowadays, the synthesis of nitrogen containing heterocycles is an important direction in the fields of pesticidal chemistry [1–3], medicinal chemistry [4], polymer chemistry [5], coordination chemistry [6] and industrial chemistry [7]. 1,2,4-Triazole derivatives and pyridine derivatives often display broad and diverse biological activities [8–10]. Some reports found that fused heterocycles generally displayed mixed properties of the corresponding single heterocycles. Many references proved that the fusing of triazole and pyridine rings was a good way to produce highly active compounds, such as herbicidal [11, 12], antifungal [13, 14], anticonvulsant [15], antibacterial activity [16]. Furthermore, the acylhydrazone structure is considered an important pharmacophore in drug discovery [17]. In the past years, there have been many reports in the literature for the synthesis and biological activities of hydrazone derivatives [18–20], such as acaricidal, anti-cancer, insecticidal, antifungal, antibacterial, antimicrobial and antileishmanial activity. In addition, hydrazones also are very useful starting materials in bioactive heterocycles, such as β-lactams, pyrazoles, and pyrazines.

**Scheme 1:**
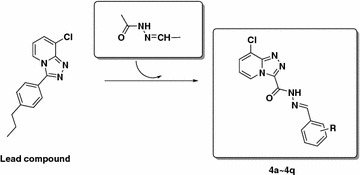
Design strategy of compounds **4a**–**4q**

In our previous work, many 1,2,4-triazolo[4,3-a]pyridine derivatives were designed and synthesized, which exhibit excellent and diverse activity [11–14]. In line with our continuous efforts to synthesize bioactive lead compounds for crop protection [21–27], the title 1,2,4-triazolo[4,3-a]pyridine derivatives were designed and synthesized by introducing acylhydrazone pharmacophore into the lead compound (Scheme 1).

## Results and discussion

### Synthesis

**Scheme 2:**
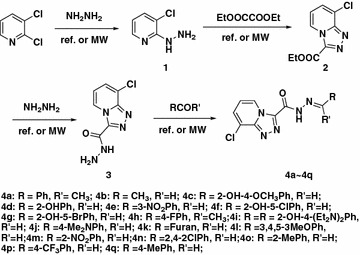
The synthetic route of compounds **4a**–**4q**

The key intermediate 8-chloro-[1, 2, 4]triazolo[4,3-a]pyridine-3-carbohydrazide was synthesized according to the Ref. [28]. Microwave technology was applied to the synthetic reaction to shorten the reaction time and increase the yield. First, the one pot synthesis of intermediate **1** under microwave irradiation was applied, but the result was not better than that of conventional condition. Then intermediate **1** was cyclized with diethyl oxalate lead to the intermediate **2** by a condensation reaction. At last, the 8-chloro-[1, 2, 4]triazolo[4,3-a]pyridine-3-carbohydrazide reacted with different aldehyde in ethanol was synthesized under microwave irradiation conditions. This reaction was completed with higher yields compared with the conventional mode of heating. The synthetic route is showed in Scheme 2.

The reaction parameters were optimized for the synthesis of title compounds. The title compounds were prepared from 8-chloro-[1, 2, 4]triazolo[4,3-a]pyridine-3-carbohydrazide and substituted aldehyde under microwave irradiation condition, leading to the desired compounds in 82–94 % yields. The compound **4d** was chosen as a model reaction under different conditions. Several key reaction conditions were studied, such as reaction temperature, reaction times, reaction mode (conventional or microwave irradiation). The results are illustrated in Table 1. From Table 1, it is indicated that the microwave irradiation method allowed a shorter reaction time, compared with the room temperature or reflux condition. Also we can see that the yield of compound **4d** is higher under microwave irradiation condition than that of room temperature or reflux condition. Under the microwave irradiation condition, the yield increase, when the reaction time is prolonged (10 min). Meanwhile, the longer reaction time held higher yield. From the Table 1, the best reaction condition is 78 °C and 10 min.

**Table 1:** Comparison of yields of **4d** through methods with or without microwave irradiation

Entry	Solvent	Method	Time (min)	Temperature/°C	Yield/ %
1	EtOH	No-MW	300	r.t.	90
2	EtOH	No-MW	120	Reflux	85
3	EtOH	MW	1	78	68
4	EtOH	MW	5	78	80
5	EtOH	MW	10	78	92
6	EtOH	MW	15	78	92
7	EtOH	MW	10	70	78
8	EtOH	MW	10	85	91

All the compounds were identified and characterized by FTIR, ^1^H NMR, ^13^C NMR, ^19^F NMR, MS and elemental analysis. In the ^1^H NMR spectra of target compounds, all the –NH proton signals of the title compounds can be found around 10–13 ppm. The appearance of signals at ~7.0, ~7.5 and ~9.3 ppm are assigned to pyridine ring. The infrared spectrum of acyl hydrozone derivatives 4 showed absorption bands at 3139–3500 cm^−1^ for N–H stretching. The characteristic stretching vibrations ν (C=O) and ν (C=N) appears at 1657–1724, 1618–1686 cm^−1^ respectively. Meanwhile, most of the title compounds exhibited the M + H^+^ peak in the ESI–MS results.

### Antifungal activities and SAR

The antifungal activities of compound **4a**–**4q** were evaluated in vivo at 100 μg/mL against *Stemphylium lycopersici* (Enjoji) Yamamoto (SL)*, Fusarium oxysporum* sp. Cucumebrium (FO) and *Botrytis cinerea* (BC) and the bioassay results were listed in Table 2. From Table 2, compound **4i**(82.74 %) and **4k**(83.53 %) possessed good activity against SL, much better than that of control zhongshengmycin (59.58 %). Among the other, compound **4h**(63.99 %), **4l**(62.30 %), **4p**(58.63 %), **4q**(61.61 %) exhibited good effect against SL, they displayed a comparable level of activity as the control zhongshengmycin. For FO, compound **4k** exhibited excellent effect (88.89 %), better than that of thiophanate-methyl(81.69 %). Meanwhile, compounds **4a**, **4b**, **4e**, **4l**, **4m**, **4n**, **4o**, **4p** and **4q** showed moderate effect against FO with the inhibitory values of 53.89, 75.56, 64.44, 77.22, 70.00, 66.67, 50.00, 69.81, 65.56 % respectively. Unfortunately, most of the compounds had low antifungal activities against *Botrytis cinerea*.

**Table 2:** The antifungal activity of title compounds in vivo at 100 μg/mL

No.	R	*Stemphylium lycopersici*	*Fusarium oxysporum*	*Botrytis cinerea*
**4a**		7.14	53.89	4.44
**4b**		2.38	75.56	10.00
**4c**		2.83	38.61	12.22
**4d**		40.18	46.67	22.22
**4e**		45.54	64.44	23.33
**4f**		39.88	13.33	21.11
**4g**		26.19	16.67	29.63
**4h**		63.99	31.11	20.00
**4i**		82.74	22.22	24.44
**4j**		69.05	4.44	21.11
**4k**		83.53	88.89	16.67
**4l**		62.30	77.22	20.00
**4m**		39.29	70.00	19.44
**4n**		26.19	66.67	23.33
**4o**		37.20	50.00	20.00
**4p**		58.63	69.81	11.11
**4q**		61.61	65.56	24.44
Zhongshengmycin		59.58		
Thiophanate-methyl			81.69	
Cyprodynil				45.56

From Table 2, the preliminary structure and activity relationship (SAR) analysis indicated that compound with electron donating group at *para* position of benzene ring exhibited significant antifungal activity against SL. For example, compound **4h**(*p*-F), **4l**(*p*-N(CH_3_)_2_), **4p**(*p*-CF_3_) and **4q**(*p*-CH_3_) displayed >50 % inhibitory activities. Also we found that the five-membered ring (Furan ring) held better activity against SL and FO than that of alkyl or aryl group. For the substituted salicylaldehydes, only compound **4i** exhibited excellent antifungal activity against SL. On the other hand, single or poly substituted compounds on the benzene ring both showed good activity against FO.

### DFT calculation and SAR

In order to study their structure-active relationship, we choose a highly active compound **4k** and low activity compound **4c** as model compounds; the frontier orbitals and LogP were calculated. The LogP, energy of HOMO and LUMO, total energy and energy gap are listed in Table 3.

**Table 3:** LogP, total energy, energy gap and frontier orbital energy

DFT	**4c**	**4k**
*E*_total_/Hartree^b^	−1519.50133881	−1339.28590225
*E*_HOMO_/Hartree	−0.12708	−0.23503
*E*_LUMO_/Hartree	0.00908	−0.07634
Δ*E* ^a^/Hartree	0.13616	0.15869
LogP	1.91	−0.43

^a^Δ*E* = *E*_LUMO_ − *E*_HOMO_

^b^1 Hartree = 4.35974417 × 10^−18^, J = 27.2113845 eV

**Fig. 1:**
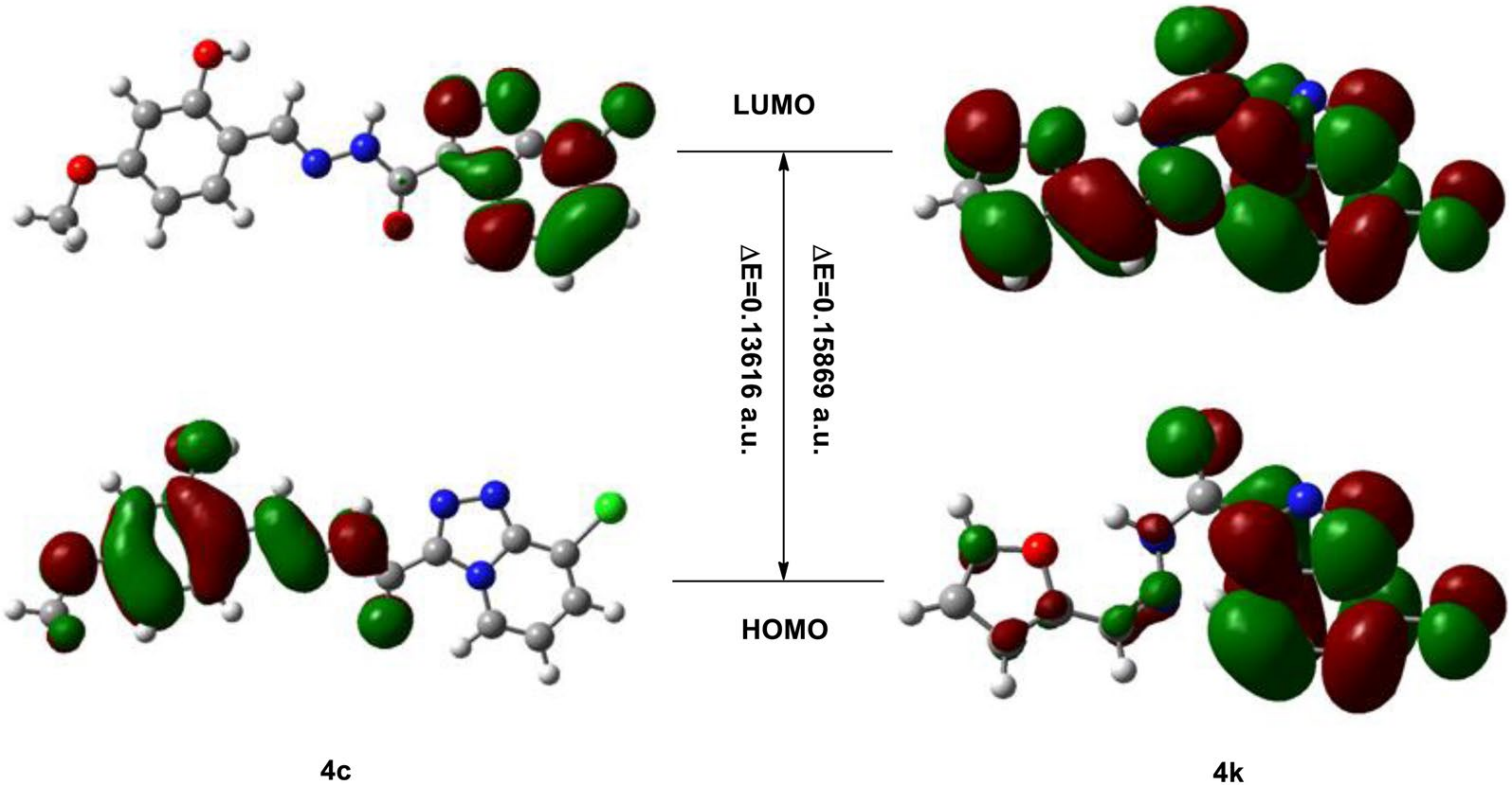
Frontier molecular orbitals of compound **4c** and **4k**

According to the frontier molecular orbital theory, HOMO has the priority to provide electrons, while LUMO can accept electrons firstly [29, 30]. As we can see from Fig. 1, the LUMO and HOMO are different between the high active compound **4k** and low active compound **4c**, especially in the orient of electron transition and energy gap. For the HOMO, the electron of compound **4k** is mainly concentrated on the fused 1,2,4-triazolo[4,3-a]pyridine ring and a little on the acyl hydrazine bridge and furan ring, while for the compound **4c**, the electron is mainly concentrated on the acyl hydrazine bridge and phenyl ring, but the fused 1,2,4-triazolo[4,3-a]pyridine ring had no electrons. As for the LUMO, The electron of compound **4k** is evenly distributed among the 1,2,4-triazolo[4,3-a]pyridine ring, acyl hydrazone group and furan ring. But the electron of compound **4c** is located on the 1,2,4-triazolo[4,3-a]pyridine ring. The possible reasons of different antifungal activity between the compound **4c** and **4k** is electron transition direction and energy gap. From Fig. 1, we assumed that the compound with higher energy gap exhibited higher antifungal activity. Also the 1,2,4-triazolo[4,3-*a*]pyridine ring is important for the higher active compound. The other impact fact is LogP. From Table 1, the LogP is different between the two compounds.

**Fig. 2:**
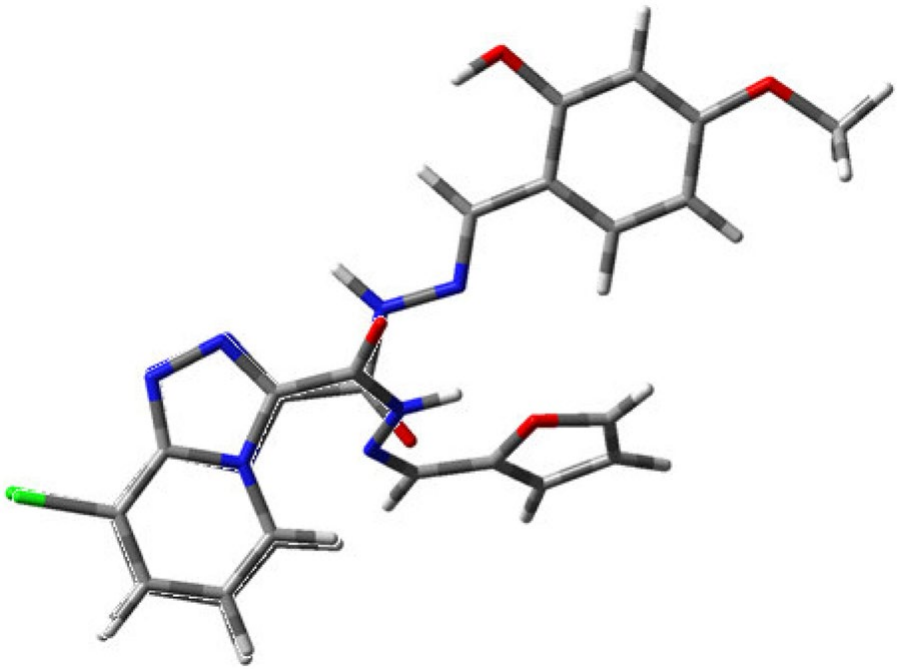
Overlay of energy-minimized structures of **4c** and **4k**

The optimized structures of the compound **4c** and **4k** are presented in Fig. 2. From Fig. 2, we can find that the orientations of amide groups are different. As we known, the conformation of compound is important for the biological activity due to the bind mode between the receptor and acceptor. So we speculate that the conformation of highly active compound is perpendicular between the 1,2,4-triazolo[4,3-a]pyridine ring and the aromatic ring. Otherwise, when the conformation of low active compound, the aromatic ring is parallel with the 1,2,4-triazolo[4,3-a]pyridine ring. These important clues will be helpful in the design of more potent compounds in the future.

## Methods

### Instruments

All the chemical reagents are analytical grade or prepared by our lab. Melting points were measured using an X-4 apparatus and were uncorrected. ^1^H NMR spectra were recorded on a Bruker Avance 500 MHz spectrometer using DMSO-d_6_ as solvent. ^13^C NMR and ^19^F NMR spectra were recorded on a Bruker Avance 600 MHz spectrometer using DMSO-d_6_ as solvent. Mass spectra were determined on a Thermo Finnigan LCQ Advantage LC/mass detector instrument. Elemental analysis data of title compounds were collected by a Perkin-Elmer 240C analyzer. CEM Discover Focused Synthesizer was used to carry out the microwave reaction (600 W, 2450 MHz).

### Synthesis

The key intermediate 1, 2, 3 are synthesized according to our previous work [28]. The title compounds **4a**–**4q** was synthesized from the intermediate **3** and different aldehydes or ketones in the solution of ethanol at the condition of microwave (150 w, 78 °C, 200 psi, 10 min). All the other compounds are synthesized according to the procedure (Scheme 2).

*8*-*Chloro*-*N′*-*(1*-*phenylethylidene)*-*[1,2,4]triazolo[4,3*-*a]pyridine*-*3*-*carbohydrazide* (**4a**) white yellow crystal, yield 82 %, m.p. > 300 °C; FT-IR (KBr, cm^−1^): ν 3416, 3216, 3128, 1690, 1662, 1539, 1478, 1382, 1342, 1258, 1217, 1088, 948, 860, 794; ^1^H NMR (DMSO-d_6_, 500 MHz), δ: 2.51(s, 3H, CH_3_), 7.08(t, *J* = 7.0 Hz, 1H, Py), 7.46(s, 3H, Ar), 7.56(d, *J* = 7.5 Hz, 1H, Py), 7.94(s, 2H, Ar), 9.38(d, *J* = 7.0 Hz, 1H, Py), 10.35(s, 1H, NH); ^13^C NMR (150 MHz, DMSO-d_6_) δ 15.19, 116.52, 120.83, 125.56, 125.80, 127.11, 128.95, 129.26, 129.39, 138.06, 139.91, 141.37, 149.04, 156.91, 168.78; MS (ESI), m/z: 314(M+1)^+^. Elemental anal. For C_15_H_12_ClN_5_O (%), calculated: C, 57.42; H, 3.86; N, 22.32; found: C, 57.65; H, 3.76; N, 22.51.

*8*-*Chloro*-*N′*-*ethylidene*-*[1,2,4]triazolo[4,3*-*a]pyridine*-*3*-*carbohydrazide* (**4b**) white yellow crystal, yield 92 %, m.p. > 300 °C; FT-IR (KBr, cm^−1^): ν 3219, 3116, 2995, 1724, 1686, 1578, 1491, 1239, 1109, 1039, 855, 794, 688, 535; ^1^H NMR (DMSO-d_6_, 500 MHz), δ: 1.95(s, 3H, CH_3_), 7.24(t, *J* = 7.2 Hz, 1H, Py), 7.82(d, *J* = 7.3 Hz, 1H, Py), 9.11(d, *J* = 7.0 Hz, 1H, Py), 10.03(s, 1H, CH), 11.02(s, 1H, NH); ^13^C NMR (150 MHz, DMSO-d_6_) δ 21.04, 116.52, 120.82, 125.55, 129.25, 139.90, 149.03, 156.90, 168.78; MS (ESI), m/z: 238(M+1)^+^. Elemental anal. For C_9_H_8_ClN_5_O (%), calculated: C, 45.49; H, 3.39; N, 29.47; found: C, 45.55; H, 3.21; N, 29.65.

***8***-*Chloro*-*N′*-*(2*-*hydroxy*-*4*-*methoxybenzylidene)*-*[1,2,4]triazolo[4,3*-*a]pyridine*-*3*-*carbohydrazide* (**4c**) white yellow crystal, yield 90 %, m.p. > 300 °C; FT-IR (KBr, cm^−1^): ν 3500, 3141, 3083, 1709, 1677, 1631, 1606, 1508, 1457, 1251, 1230, 1204, 1170, 1092, 1022, 965, 832, 742; ^1^H NMR (DMSO-d_6_, 500 MHz), δ: 3.79(s, 3H, OCH_3_), 6.55(d, *J* = 9.0 Hz, 1H, Ar), 7.25(t, *J* = 7.2 Hz, 1H, Py), 7.43(d, *J* = 8.5 Hz, 1H, Py), 7.83(d, *J* = 7.3 Hz, 1H, Ar), 8.74(s, 1H, Ar), 9.22(d, *J* = 7.0 Hz, 1H, Py), 11.49(s, 1H, CH), 11.97(s, 1H, NH), 13.01(s, 1H, OH); ^13^C NMR (150 MHz, DMSO-d_6_) δ 55.84, 101.69, 107.15, 112.19, 116.42, 120.78, 125.92, 129.38, 131.79, 140.42, 149.14, 150.93, 153.82, 160.03, 162.86; MS (ESI), m/z: 346(M+1)^+^. Elemental anal. For C_15_H_12_ClN_5_O_3_ (%), calculated: C, 52.11; H, 3.50; N, 20.26; found: C, 51.98; H, 3.44; N, 20.43.

*8*-*Chloro*-*N′*-*(2*-*hydroxybenzylidene)*-*[1,2,4]triazolo[4,3*-*a]pyridine*-*3*-*carbohydrazide* (**4d**) white yellow crystal, yield 92 %, m.p. > 300 °C; FT-IR (KBr, cm^−1^): ν 3178, 3143, 3052, 1667, 1619, 1549, 1486, 1445, 1354, 1271, 1271, 1238, 1220, 1096, 1039, 954, 848, 762; ^1^H NMR (DMSO-d_6_, 500 MHz), δ: 6.92–6.96(m, 2H, Ar), 7.23–7.33(m, 2H, Ar), 7.54(t, *J* = 7.2 Hz, 1H, Py), 7.82(s, 1H, CH), 8.28(d, *J* = 7.3 Hz, 1H, Py), 9.21(d, *J* = 7.0 Hz, 1H, Py), 11.13(s, 1H, NH), 13.10(s, 1H, OH); ^13^C NMR (150 MHz, DMSO-d_6_) δ 116.48, 116.98, 119.15, 119.94, 120.79, 125.93, 129.43, 130.02, 132.25, 140.40, 149.17, 150.41, 154.08, 159.03; MS (ESI), m/z: 316(M+1)^+^. Elemental anal. For C_14_H_10_ClN_5_O_2_ (%), calculated: C, 53.26; H, 3.19; N, 22.18; found: C, 53.35; H, 3.22; N, 22.41.

*8*-*Chloro*-*N′*-*(3*-*nitrobenzylidene)*-*[1,2,4]triazolo[4,3*-*a]pyridine*-*3*-*carbohydrazide* (**4e**) white yellow crystal, yield 94 %, m.p. > 300 °C; FT-IR (KBr, cm^−1^): ν 3322, 3158, 1683, 1620, 1533, 1486, 1451, 1353, 1275, 1214, 1146, 1078, 959, 897, 853, 788, 736; ^1^H NMR (DMSO-d_6_, 500 MHz), δ: 7.27(t, *J* = 7.0 Hz, 1H, Py), 7.73(t, *J* = 7.8 Hz, 1H, Ar), 7.84–7.87(m, 2H, Py & Ar), 8.10 ~ 8.17(m, 2H, Ar), 9.08(s, 1H, CH), 9.21(d, *J* = 6.8 Hz, 1H, Py), 13.21(s, 1H, NH); ^13^C NMR (150 MHz, DMSO-d_6_) δ 116.58, 120.83, 121.57, 125.08, 125.87, 129.48, 131.09, 134.06, 136.39, 140.48, 147.71, 148.75, 154.48, 162.17; MS (ESI), m/z: 345(M+1)^+^. Elemental anal. For C_14_H_9_ClN_6_O_3_ (%), calculated: C, 48.78; H, 2.63; N, 24.38; found: C, 48.86; H, 2.76; N, 24.99.

*8*-*Chloro*-*N′*-*(5*-*chloro*-*2*-*hydroxybenzylidene)*-*[1,2,4]triazolo[4,3*-*a]pyridine*-*3*-*carbohydrazide* (**4f**) white yellow crystal, yield 88 %, m.p. > 300 °C; FT-IR (KBr, cm^−1^): ν 3246, 3135, 1676, 1618, 1521, 1477, 1458, 1342, 1267, 1211, 1184, 1145, 1084, 846, 724; ^1^H NMR (DMSO-d_6_, 500 MHz), δ: 6.98(d, *J* = 8.7 Hz, 1H, Ar), 7.28(t, *J* = 7.2 Hz, 1H, Py), 7.35(d, *J* = 8.5 Hz, 1H, Py), 7.68(s, 1H, Ar), 7.93(d, *J* = 7.4 Hz, 1H, Ar), 8.82(s, 1H, CH), 9.21(d, *J* = 6.9 Hz, 1H, Py), 11.05(s, 1H, NH), 13.16(s, 1H, OH); ^13^C NMR (150 MHz, DMSO-d_6_) δ116.51, 118.80, 120.80, 121.29, 123.54, 125.92, 127.86, 129.45, 131.62, 140.41, 147.80, 149.18, 154.25, 156.59; MS (ESI), m/z: 350(M+1)^+^. Elemental anal. For C_14_H_9_Cl_2_N_5_O_2_ (%), calculated: C, 48.02; H, 2.59; N, 20.00; found: C, 47.80; H, 2.75; N, 20.21.

*N′*-*(5*-*bromo*-*2*-*hydroxybenzylidene)*-*8*-*chloro*-*[1,2,4]triazolo[4,3*-*a]pyridine*-*3*-*carbohydrazide* (**4g**) white yellow crystal, yield 90 %, m.p. > 300 °C; FT-IR (KBr, cm^−1^): ν 3249, 3134, 1674, 1619, 1613, 1519, 1473, 1455, 1341, 1266, 1210, 1182, 1077, 958, 846, 742; ^1^H NMR (DMSO-d_6_, 500 MHz), δ: 6.92(d, *J* = 8.7 Hz, 1H, Ar), 7.27(t, *J* = 7.2 Hz, 1H, Py), 7.45(d, *J* = 8.5 Hz, 1H, Py), 7.80(s, 1H, Ar), 7.84(d, *J* = 7.4 Hz, 1H, Ar), 8.82(s, 1H, CH), 9.21(d, *J* = 6.9 Hz, 1H, Py), 11.10(s, 1H, NH), 13.15(s, 1H, OH); ^13^C NMR (150 MHz, DMSO-d_6_) δ 116.58, 120.83, 121.57, 125.08, 125.87, 129.48, 131.09, 134.06, 136.39, 140.48, 147.71, 148.75, 149.21, 154.48; MS (ESI), m/z: 395(M+1)^+^. Elemental anal. For C_14_H_9_BrClN_5_O_2_ (%), calculated: C, 42.61; H, 2.30; N, 17.75; found: C, 42.45; H, 2.25; N, 17.71.

*8*-*Chloro*-*N′*-*(1*-*(4*-*fluorophenyl)ethylidene)*-*[1,2,4]triazolo[4,3*-*a]pyridine*-*3*-*carbohydrazide* (**4h**) white yellow crystal, yield 93 %, m.p. > 300 °C; FT-IR (KBr, cm^−1^): ν 3345, 3223, 3137, 1698, 1662, 1538, 1504, 1497, 1382, 1342, 1217, 1158, 1089, 949, 858, 794, 742; ^1^H NMR (DMSO-d_6_, 500 MHz), δ: 2.47(s, 3H, CH_3_), 7.05-7.13(m, 3H, Ar and Py), 7.54(t, *J* = 7.2 Hz, 1H, Py), 7.90–7.93(m, 2H, Ar), 9.35(d, *J* = 6.9 Hz, 1H, Py), 10.31(s, 1H, NH); ^13^C NMR (150 MHz, DMSO-d_6_) δ 15.23, 115.80, 115.93, 116.51, 120.83, 125.55, 125.78, 129.25, 129.40, 139.91, 149.04, 156.91, 168.78; ^19^F NMR (564 MHz, DMSO-d_6_) δ -111.38; MS (ESI), m/z: 332(M+1)^+^. Elemental anal. For C_15_H_11_ClFN_5_O (%), calculated: C, 54.31; H, 3.34; N, 21.11; found: C, 54.18; H, 3.52; N, 21.31.

*8*-*Chloro*-*N′*-*(4*-*(diethylamino)*-*2*-*hydroxybenzylidene)*-*[1,2,4]triazolo[4,3*-*a]pyridine*-*3*-*carbohydrazide* (**4i**) white yellow crystal, yield 92 %, m.p. >300 °C; FT-IR (KBr, cm^−1^): ν 3212, 2970, 2931, 1676, 1629, 1596, 1518, 1488, 1350, 1247, 1132, 1078, 1040, 850, 758, 738; ^1^H NMR (DMSO-d_6_, 500 MHz), δ: 1.18–1.25(m, 6H, 2CH_3_), 3.36-3.42(m, 4H, 2CH_2_), 6.62(m, 2H, Ar), 7.03–7.10(m, 2H, Py and Ar), 7.52(d, *J* = 7.2 Hz, 1H, Py), 8.25(s, 1H, Ar), 8.45(s, 1H, CH), 9.34(d, *J* = 7.1 Hz, 1H, Py), 10.22(s, 1H, NH); ^13^C NMR (150 MHz, DMSO-d_6_) δ 13.02, 44.30, 97.93, 104.31, 106.31, 116.30, 120.75, 125.92, 129.25, 132.37, 140.55, 149.06, 150.92, 152.06, 153.35, 160.35; MS (ESI), m/z: 387(M+1)^+^. Elemental anal. For C_18_H_19_ClN_6_O_2_ (%), calculated: C, 55.89; H, 4.95; N, 21.73; found: C, 55.99; H, 4.76; N, 21.69.

*8*-*Chloro*-*N′*-*(4*-*(dimethylamino)benzylidene)*-*[1,2,4]triazolo[4,3*-*a]pyridine*-*3*-*carbohydrazide* (**4j**) white yellow crystal, yield 90 %, m.p. 280–281 °C; FT-IR (KBr, cm^−1^): ν 3487, 1674, 1596, 1525, 1466, 1367, 1255, 1189, 1087, 809, 740; ^1^H NMR (DMSO-d_6_, 500 MHz), δ: 2.99(s, 6H, 2CH_3_), 6.78(d, *J* = 8.8 Hz, 2H, Ar), 7.24(t, *J* = 7.2 Hz, 1H, Py), 7.54(d, *J* = 8.7 Hz, 1H, Ar), 7.81(d, *J* = 7.2 Hz, 1H, Py), 8.49(s, 1H, CH), 9.21(d, *J* = 6.9 Hz, 1H, Py), 12.50(s, 1H, NH); ^13^C NMR (150 MHz, DMSO-d_6_) δ 40.22, 112,25, 116.26, 120.75, 121.72, 125.86, 129.18, 138.76, 140.77, 149.03, 150.89, 152.21, 153.69; MS (ESI), m/z: 343(M+1)^+^. Elemental anal. For C_16_H_15_ClN_6_O (%), calculated: C, 56.06; H, 4.41; N, 24.52; found: C, 55.89; H, 4.47; N, 24.46.

*8*-*Chloro*-*N′*-*(furan*-*2*-*ylmethylene)*-*[1,2,4]triazolo[4,3*-*a]pyridine*-*3*-*carbohydrazide* (**4k**) white yellow crystal, yield 91 %, m.p. 278–279 °C; FT-IR (KBr, cm^−1^): ν 3269, 3160, 3065, 1666, 1626, 1541, 1479, 1349, 1298, 1220, 1156, 1081, 1012, 935, 851, 803, 732; ^1^H NMR (DMSO-d_6_, 500 MHz), δ: 6.93(d, 2H, Furan), 7.07(t, *J* = 6.9 Hz, 1H, Py), 7.54–7.57(m, 2H, Py and Furan), 8.32(s, 1H, CH), 9.33(d, *J* = 6.9 Hz, 1H, Py), 11.20(s, 1H, NH); ^13^C NMR (150 MHz, DMSO-d_6_) δ 112.84, 114.79, 116.44, 120.77, 125.91, 129.36, 139.57, 140.54, 146.06, 149.13, 149.74, 154.12; MS (ESI), m/z: 290(M+1)^+^. Elemental anal. For C_12_H_8_ClN_5_O_2_ (%), calculated: C, 49.75; H, 2.78; N, 24.18; found: C, 49.68; H, 2.76; N, 24.44.

*8*-*Chloro*-*N′*-*(3,4,5*-*trimethoxybenzylidene)*-*[1,2,4]triazolo[4,3*-*a]pyridine*-*3*-*carbohydrazide* (**4l**) white yellow crystal, yield 88 %, m.p. > 300 °C; FT-IR (KBr, cm^−1^): ν 3139, 2994, 1657, 1629, 1569, 1519, 1346, 1246, 1226, 1066, 913, 850, 739; ^1^H NMR (DMSO-d_6_, 500 MHz), δ: 3.96(s, 9H, 3OCH_3_), 7.04(s, 2H, Ar), 7.28(t, *J* = 7.0 Hz, 1H, Py), 7.84(d, *J* = 6.9 Hz, 1H, Py), 8.55(s, 1H, CH), 9.21(d, *J* = 7.2 Hz, 1H, Py), 12.83(s, 1H, NH); ^13^C NMR (150 MHz, DMSO-d_6_) δ 56.46, 60.62, 104.95, 116.44, 120.80, 125.85, 129.34, 130.05, 139.98, 139.98, 140.60, 149.13, 153.70, 154.16; MS (ESI), m/z: 391(M+1)^+^. Elemental anal. For C_17_H_16_ClN_5_O_4_ (%), calculated: C, 52.38; H, 4.14; N, 17.97; found: C, 52.51; H, 4.28; N, 8.21.

*8*-*Chloro*-*N′*-*(2*-*nitrobenzylidene)*-*[1,2,4]triazolo[4,3*-*a]pyridine*-*3*-*carbohydrazide* (**4m**) white yellow crystal, yield 90 %, m.p. >300 °C; FT-IR (KBr, cm^−1^): ν 3290, 1679, 1628, 1584, 1536, 1470, 1452, 1361, 1226, 1211, 1153, 1094, 1049, 914, 843, 743; ^1^H NMR (DMSO-d_6_, 500 MHz), δ: 7.27(t, *J* = 7.0 Hz, 1H, Py), 7.72(t, *J* = 7.8 Hz, 1H, Py), 7.84–7.87(m, 2H, Ar), 8.10-8.17(m, 2H, Ar), 9.08(s, 1H, CH), 9.21(d, *J* = 6.8 Hz, 1H, Py), 12.21(s, 1H, NH); ^13^C NMR (150 MHz, DMSO-d_6_) δ 116.52, 120.81, 125.19, 125.89, 128.63, 128.98, 129.46, 131.50, 134.29, 140.51, 145.52, 148.85, 149.19, 154.54; MS (ESI), m/z: 345(M+1)^+^. Elemental anal. For C_14_H_9_ClN_6_O_3_ (%), calculated: C, 48.78; H, 2.63; N, 24.38; found: C, 48.95; H, 2.45; N, 24.43.

*8*-*Chloro*-*N′*-*(2,4*-*dichlorobenzylidene)*-*[1,2,4]triazolo[4,3*-*a]pyridine*-*3*-*carbohydrazide* (**4n**) white yellow crystal, yield 91 %, m.p. >300 °C; FT-IR (KBr, cm^−1^): ν 3323, 3158, 3082, 1684, 1619, 1532, 1487, 1451, 1353, 1214, 1146, 1078, 1048, 958, 897, 853, 788, 735, 694; ^1^H NMR (DMSO-d_6_, 500 MHz), δ: 7.27(t, *J* = 7.0 Hz, 1H, Py), 7.53(d, *J* = 8.3 Hz, 1H, Ar), 7.74(s, 1H, Ar), 7.85(d, *J* = 7.2 Hz, 1H, Py), 8.03(d, *J* = 8.5 Hz, 1H, Ar), 9.05(s, 1H, CH), 9.21(d, *J* = 6.9 Hz, 1H, Py), 12.13(s, 1H, NH); ^13^C NMR (150 MHz, DMSO-d_6_) δ 116.55, 120.83, 125.88, 128.58, 128.64, 129.46, 129.97, 131.09, 134.72, 135.92, 140.49, 145.14, 149.20, 154.40; MS (ESI), m/z: 369(M+1)^+^. Elemental anal. For C_14_H_8_Cl_3_N_5_O (%), calculated: C, 45.62; H, 2.19; N, 19.00; found: C, 45.65; H, 2.33; N, 19.21.

*8*-*Chloro*-*N′*-*(2*-*methylbenzylidene)*-*[1,2,4]triazolo[4,3*-*a]pyridine*-*3*-*carbohydrazide* (**4o**) white yellow crystal, yield 86 %, m.p. >300 °C; FT-IR (KBr, cm^−1^): ν 3222, 3060, 1680, 1598, 1548, 1523, 1488, 1454, 1359, 1234, 1222, 1112, 1085, 1039, 949, 849, 784, 742, 693; ^1^H NMR (DMSO-d_6_, 500 MHz), δ: 2.37(s, 3H, CH_3_), 7.24–7.28(m, 1H, Ar), 7.37(t, *J* = 7.5 Hz, 1H, Py), 7.53(d, *J* = 7.5 Hz, 1H, Ar), 7.57(s, 1H, Ar), 7.73(d, *J* = 7.2 Hz, 1H, Py), 7.84(d, *J* = 7.2 Hz, 1H, Py), 8.51(s, 1H, CH), 9.21(d, *J* = 6.9 Hz, 1H, Py), 12.91(s, 1H, NH); ^13^C NMR (150 MHz, DMSO-d_6_) δ 21.35, 116.43, 120.79, 125.15, 125.88, 128.06, 129.29, 129.33, 131.62, 134.52, 138.66, 140.59, 149.13, 150.21, 154.21; MS (ESI), m/z: 315(M+1)^+^. Elemental anal. For C_15_H_12_ClN_5_O (%), calculated: C, 57.42; H, 3.86; N, 22.32; found: C, 57.38; H, 4.01; N, 22.11.

*8*-*Chloro*-*N′*-*(4*-*(trifluoromethyl)benzylidene)*-*[1,2,4]triazolo[4,3*-*a]pyridine*-*3*-*carbohydrazide* (**4p**) white yellow crystal, yield 92 %, m.p. >300 °C; FT-IR (KBr, cm^−1^): ν 3321, 3153, 1673, 1620, 1545, 1523, 1487, 1449, 1331, 1297, 1216, 1153, 1116, 1069, 1018, 952, 838, 794, 743; ^1^H NMR (DMSO-d_6_, 500 MHz), δ: 7.28(t, *J* = 7.2 Hz, 1H, Py), 7.82–7.85(m, 3H, Ar and Py), 7.97(d, *J* = 8.0 Hz, 2H, Ar) 8.72(s, 1H, CH), 9.21(d, *J* = 6.9 Hz, 1H, Py), 13.02(s, 1H, NH); ^13^C NMR (150 MHz, DMSO-d_6_) δ 116.55, 120.82, 125.89, 126.21, 126.31, 128.36, 129.46, 138.53, 140.51, 148.32, 148.36, 149.20, 154.44; ^19^F NMR (564 MHz, DMSO-d_6_) δ -61.19; MS (ESI), m/z: 368(M+1)^+^. Elemental anal. For C_15_H_9_ClF_3_N_5_O (%), calculated: C, 48.99; H, 2.47; N, 19.05; found: C, 49.21; H, 2.72; N, 18.88.

*8*-*Chloro*-*N′*-*(4*-*methylbenzylidene)*-*[1,2,4]triazolo[4,3*-*a]pyridine*-*3*-*carbohydrazide* (**4q**) white yellow crystal, yield 88 %, m.p. >300 °C; FT-IR (KBr, cm^−1^): ν 3294, 3143, 1694, 1671, 1605, 1541, 1509, 1488, 1459, 1357, 1233, 1220, 1152, 1074, 1043, 953, 910, 842, 813, 743; ^1^H NMR (DMSO-d_6_, 500 MHz), δ: 2.36(s, 3H, CH_3_), 6.68(t, *J* = 7.5 Hz, 1H, Py), 7.65(d, *J* = 7.5 Hz, 2H, Ar), 7.85(d, *J* = 7.4 Hz, 2H, Ar), 8.07(d, *J* = 4.4 Hz, 1H, Py), 8.51(s, 1H, CH), 9.21(d, *J* = 6.9 Hz, 1H, Py), 12.78(s, 1H, NH); ^13^C NMR (150 MHz, DMSO-d_6_) δ 21.55, 116.41, 120.78, 125.88, 127.77, 129.32, 130.01, 131.06, 140.62, 140.81, 149.12, 150.17, 154.15; MS (ESI), m/z: 314(M+1)^+^. Elemental anal. For C_15_H_12_ClN_5_O (%), calculated: C, 57.42; H, 3.86; N, 22.32; found: C, 57.65; H, 3.03; N, 22.55.

### Antifungal activity

Antifungal activities of compounds **4a–4q** against *Stemphylium lycopersici* (Enjoji) Yamamoto*, Fusarium oxysporum.* sp. Cucumebrium and *Botrytis cinerea* were determined according to our previous work [31]. The potted plants cucumber and tomato were used. The determine concentration of control zhongshengmycin, thiophanate-methyl, cyprodinil and the title compounds is 100 μg/mL. The three fungals *Stemphylium lycopersici* (Enjoji) Yamamoto, *Fusarium oxysporum.* sp. Cucumebrium and *Botrytis cinerea* were inoculated when the cucumber or tomato is at the stage of two seed leaves. The relative control efficacy of compounds comparing to the blank assay was calculated via the following equation:

$$ {\text{Relative control efficacy }}\left( \% \right) = \left( {CK - PT} \right)/CK \times 100\,\% $$

where CK is the average disease index during the blank assay and PT is the average disease index after treatment during testing. All experiments were replicated three times.

### Therotical calculations

The theoretical calculation was carried out using DFT methods. The geometry optimization of compound **4c** and **4k** was carried out at the B3LYP/6-31G level. The energies of HOMO, LUMO and total energy, energy gap are calculated. All these are carried out using the Gaussian 03 package [32] on the dell computer. The LogP was calculated by Chemdraw 7.0.

## Conclusions

In conclusion, a series of novel 1,2,4-triazolo[4,3-a]pyridine derivatives containing hydrazone moiety have been designed by bio-rational method based on the former lead compound by us. Many compounds were found to show good antifungal activity. The further comprehensive structure-active relationship was described by using theoretical calculation method. Among them, compound **4k** possessed excellent antifungal activities against *Stemphylium lycopersici* (Enjoji) Yamamoto and *Fusarium oxysporum.* sp. Cucumebrium.
